# Impacted lower third molars and distal caries in the mandibular second molar. Is prophylactic removal of lower third molars justified?

**DOI:** 10.4317/jced.53919

**Published:** 2017-06-01

**Authors:** José Marques, Marta Montserrat-Bosch, Rui Figueiredo, Miguel-Angel Vilchez-Pérez, Eduard Valmaseda-Castellón, Cosme Gay-Escoda

**Affiliations:** 1 DDS, MS. Master of Oral Surgery and Implantology. Professor of the Oral Surgery and Implantology Master’s Degree program. School of Dentistry, University of Barcelona (Spain); 2 DDS. Master of Oral Surgery and Implantology. School of Dentistry, University of Barcelona. Barcelona (Spain); 3 DDS, MS, PhD. Master of Oral Surgery and Implantology. Professor of the Oral Surgery and Implantology Master’s Degree program. School of Dentistry, University of Barcelona. Researcher of the IDIBELL Institute. Barcelona (Spain); 4 DDS, MS. Master of Oral Surgery and Implantology. Professor of Oral Surgery. School of Dentistry, University of Barcelona. Professor Coordinator of the Master’s in Oral Surgery and Implantology (EFHRE International University / FUCSO). Barcelona (Spain); 5 DDS, MS, PhD. Master of Oral Surgery and Implantology. Director of the Oral Surgery and Implantology Master’s Degree program. School of Dentistry, University of Barcelona. Researcher of the IDIBELL Institute. Barcelona (Spain); 6 MD, DDS, MS, PhD, EBOS, OMFS. Chairman and Full Professor of Oral and Maxillofacial Surgery. School of Dentistry, University of Barcelona. Director of the Master of Oral Surgery and Implantology (EFHRE International University / FUCSO). Coordinator Researcher of the IDIBELL Institute. Head of the Department of Oral and Maxillofacial Surgery, Teknon Medical Center. Barcelona (Spain)

## Abstract

**Background:**

The objective of this study was to evaluate the association between the presence of mandibular third molars and the occurrence of carious lesions in the distal aspect of the mandibular second molar.

**Material and Methods:**

A retrospective cohort study comprising 327 lower third molars extracted in the Oral Surgery and Implantology Master’s Degree program of the School of Dentistry of the University of Barcelona (Barcelona, Spain) was carried out. A descriptive and bivariate analysis was made. The diagnosis of caries in the second molar and the position of the mandibular third molar were evaluated through panoramic radiographies.

**Results:**

The sample included 203 patients, 94 males (46.3%) and 109 females (53.7%), with a mean age of 26,8 years and 327 lower third molars. The prevalence of second molar distal caries was 25.4% (95% CI= 20.6% to 30.2%). This pathology was significantly more frequent when the third molar was in a horizontal position (27.7%), when the contact point was at (45,8%) or below (47.0%) the cementoenamel junction (CEJ), and when the distal CEJ of the mandibular second molar and the mesial CEJ of the third molar was 7 to 12 mm apart.

**Conclusions:**

Horizontal lower third molars with contact points at or below the CEJ are more likely to produce distal caries in the mandibular second molars. Due to the high prevalence of this pathology (20.6% to 30.2%), a prophylactic removal of lower third molars with the above-mentioned features might be advisable.

** Key words:**Second molar, caries, third molar, prophylactic removal.

## Introduction

The removal of impacted lower third molars (L3M) is one of the most frequent procedures in Oral Surgery and several papers have addressed the main indications for these extractions (1,2). The decision to remove L3M associated with a pathology is often straightforward, but the necessity and validity of prophylactic third molar removal has been questioned by many investigators (1-7).

Unerupted L3M have been associated with various symptoms and pathologies, such as pericoronitis, pain and swelling, cheek ulcerations, odontogenic cysts, benign or malignant tumors, and systemic infections, among others. These teeth may also affect the adjacent second molars (L2M) producing distal caries, periodontal defects and root resorptions. However, reports have shown that a significant percentage of impacted L3M may remain free of pathology for a long period of time (4,8-10).

Nerve injuries and periodontal complications of the L2M after L3M removal have been widely discussed in the literature (6,8,9,11,12). Nevertheless, few studies relate the position of the L3M with the prevalence of distal caries in the L2M. According to recently published data, partially impacted third molars with a mesioangular or horizontal inclination that are in close relation with the mandibular second molar cementoenamel junction (CEJ) present a higher risk of causing caries (13-18). This complica-tion often leads to the extraction of both teeth (19,20).

Therefore, it is essential to detect high-risk patients in order to establish a strict follow-up protocol allowing for an early diagnosis of this pathology. Moreover, and depending on the prevalence of this pathology, a debate concerning the prophylactic extraction of selected cases might be necessary (2-5,10,20).

Thus, the aim of the present study is to determine the prevalence of L2M distal caries and to detect the main risk factors, taking into consideration the position of the L3M.

## Material and Methods

A retrospective cohort study comprising 327 lower third molars extracted in the Oral Surgery and Implantology Master’s Degree program of the School of Dentistry of the University of Barcelona (Barcelona, Spain) was carried out.

The diagnosis of caries in the L2M and the position of the L3M were evaluated by a single researcher. All panoramic radiographies had a magnification of 1:1.10 and were assessed under standardized conditions.

**Figure 1:**
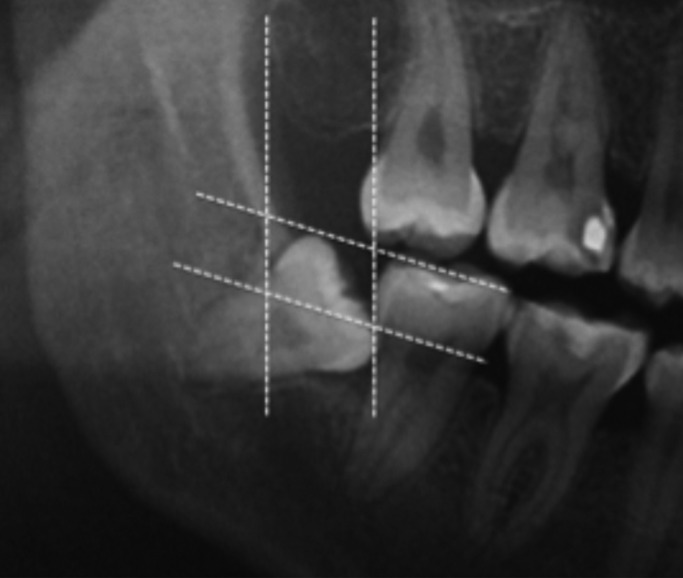
The distal space and depth of inclusion was determined by the Pell & Gregory classification method.

**Figure 2:**
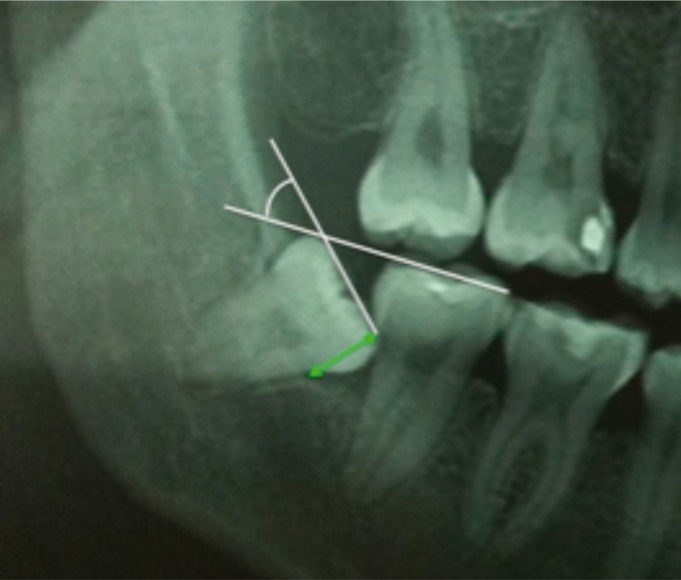
The Leone classification was applied to determine the distance between the distal CEJ of the L2M and mesial CEJ of the L3M (1-3 mm, 4-6 mm, 7-9 mm, 10-12 mm, or ≥13 mm). The angulation of the L3M was calculated by measuring the angle formed by the mandibular occlusal plane and the occlusal surface of the third molar, as described by Schiller (vertical 0°-10°, mesioangular or distoangular 11-70° and horizontal ≥71°). CEJ: cementoenamel junction; L3M: lower third molar; L2M: lower second molar.

The following data were retrieved: gender, age, number of decayed teeth, missing or filled teeth, angulation of the L3M, and distance between the distal surface of the L2M and the mesial surface of the L3M. The distal space and depth of inclusion was determined using the Pell & Gregory classification as can be observed in figure 1 (21,22). The Leone classification was applied to determine the distance between the distal CEJ of the L2M and mesial CEJ of the adjacent mandibular third molar (1-3 mm, 4-6 mm, 7-9 mm, 10-12 mm, or ≥13 mm) (Fig. 2) (23).

The angulation of the L3M was calculated by measuring the angle formed by the mandibular occlusal plane and the occlusal surface of the third molar, as described by Schiller (vertical 0°-10°, mesioangular or distoangular 11-70° and horizontal ≥71°) (24). Radiolucent pericoronal images of more than 2.5 mm were considered pathological (4).

In order to avoid interexaminer bias, a single investigator carried out the measurements. To test intra-examiner agreement, 10 patients were assessed twice and the intra-class correlation coefficient (ICC) was calculated (0,98).

The data obtained was analyzed using SPSS 19.0 statistical package (SPSS Inc., Chicago, IL, USA). When the distribution was compatible with normality, the mean and standard deviation (SD) were used. A bivariate analysis using Pearson’s chi-square and student t-tests was made. The level of significance was set at *p*<0.05 and a 95% confidence interval (95%CI) was calculated for the prevalence.

## Results

Two hundred and three patients with 327 impacted L3M were included in the study. A total of 94 males (46.3%) and 109 females (53.7%) with a mean age of 26.8 years (SD=7; range 18-45) were analyzed.

The prevalence of L2M distal caries was 25.4% (95%CI= 20.6% to 30.2%). When the L3M was horizontal, the prevalence of caries increased significantly (27.7% Vs. 13.9%) (x2=14.48; df=3; *p*=0.001). Considering the contact point, when it was at or below the CEJ, a higher number of L2M caries was observed (45.8% and 47.0% respectively versus 7.2% when the contact point was above the CEJ) (x2=27,65; df=2; *p*=0.001). This complication also increased significantly when the distal CEJ of the L2M and mesial CEJ of the adjacent L3M were 7-9 mm and 10-12 mm apart (36.7% and 36.6%, respectively; x2=18.54; df=4; *p*=0.002) ( Table 1).

**Table 1:**
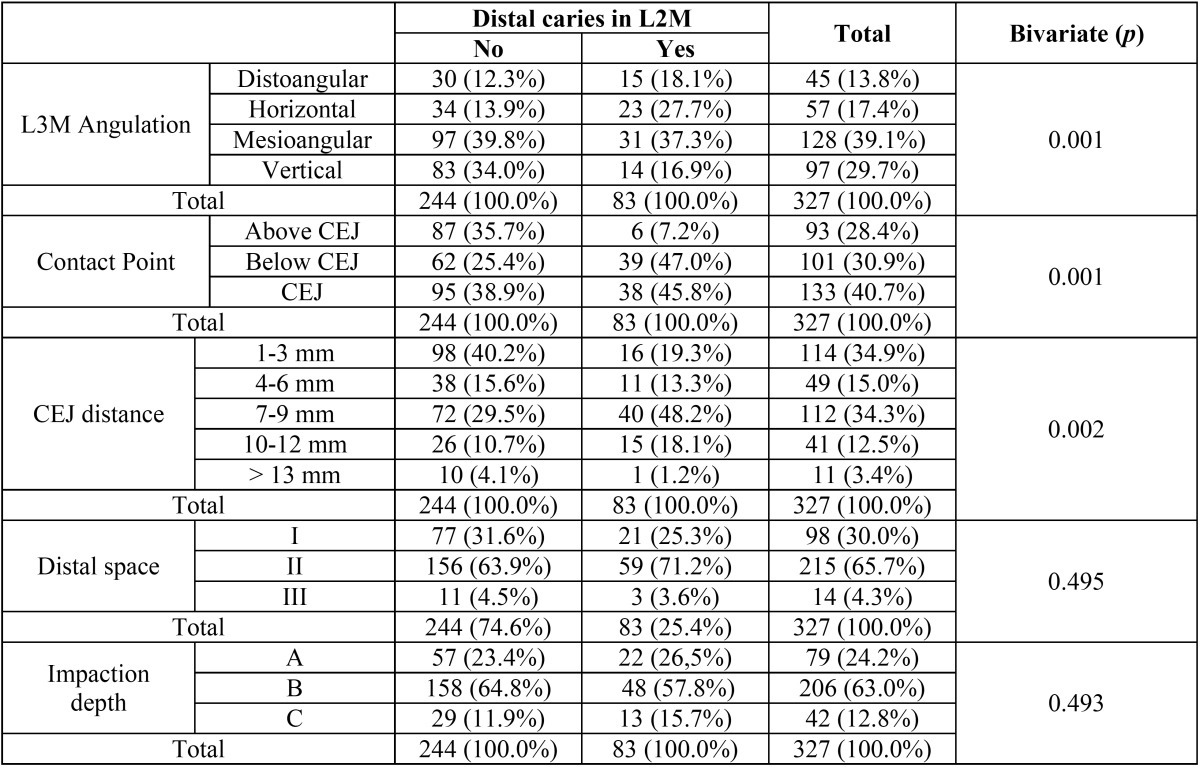
Relationship between second molar distal caries, third molar angulation, contact point, CEJ distance, distal space and impaction depth. L3M: Lower third molar. CEJ: cementoenamel junction. L2M: Lower second molar.

Age did not seem to be significantly related with development of distal caries on the L2M due to the impaction of L3M (t= 0.152; *p*=0.697).

## Discussion

Previous studies pointed out that second molars with adjacent impacted L3M have a prevalence of caries ranging from 7 to 32% (6,9,14,15,25). Authors such as Kang *et al.* (17) reported even higher figures (52%), probably due to the use of cone-beam computed tomography (CBCT) to diagnose caries. According to the present study, the prevalence of L2M distal caries ranges from 20.6 – 30.2%, confirming the findings reported by Özeç *et al.* (14), in a Turkish population (20%), and van der Linden *et al.* (32%) (26). It must be pointed out that all patients of our sample were initially evaluated by a general dental practitioner that recommended L3M extraction. Thus, it is likely that the prevalence of complications associated with the L3M in the present sample is higher than in the general population.

McArdle and Renton (27) concluded that L3M position is a more relevant factor for L2M distal caries development in comparison with other variables such as high susceptibility to dental caries in general. Regarding the angulation of the impacted tooth, most authors state that a mesioangular tilt seems to be highly associated with caries occurrence, while vertical, distoangular or ectopic impactions are unlikely to originate this pathology (6,8,13-15,17-19,25). However, the present study shows that a horizontal angulation might also be an important risk factor.

The influence of the contact point location between the second and third molars on the formation of second molar distal caries has been well documented in the literature, with similar findings to the ones of our sample (14,17). In our opinion, contact points below the CEJ are more difficult to clean, leading to higher plaque accumulation. On the other hand, third molars are associated with certain bacteria and inflammatory mediators that might enhance the development of periodontitis and caries on the adjacent teeth (13,18).

It has been suggested that the initiation and severity of distal caries in second molars increases over time, and that older patients have higher incidences of this complication (17,25). Nevertheless, the results of the present study did not show any relation between these 2 factors, probably due to the low mean age of the sample.

One of the main limitations of this study was the use of panoramic radiographies, since these have been shown to be inferior to intraoral techniques in detecting interproximal caries. Therefore, early carious lesions may not have been detected, which might have led to an underestimation in the diagnosis of L2M caries.

The development of caries in the distal aspect of the mandibular second molar not only indicates the need for L3M extraction, but also requires restorative and possible endodontic treatments of the adjacent second molar. Furthermore, L2M extraction might be necessary in cases where the carious lesion is subgingival (19). Therefore, the identification of high-risk cases is crucial and, in our opinion, the prophylactic removal of these teeth is recommended.

Horizontal or mesioangular lower third molars with contact points at or below the CEJ are more likely to produce distal caries in the mandibular second molars. Due to the high prevalence of this pathology (20.6% to 30.2%), a prophylactic removal of lower third molars with the above-mentioned features might be advisable.
